# Modeling Infectious Disease in Mice: Co-Adaptation and the Role of Host-Specific IFNγ Responses

**DOI:** 10.1371/journal.ppat.1000333

**Published:** 2009-05-29

**Authors:** Jörn Coers, Michael N. Starnbach, Jonathan C. Howard

**Affiliations:** 1 Department of Microbiology and Molecular Genetics, Harvard Medical School, Boston, Massachusetts, United States of America; 2 Institute for Genetics, University of Cologne, Cologne, Germany; The Fox Chase Cancer Center, United States of America

The advantages of the mouse as a model organism in biomedical research are many. The molecular and genetic toolbox developed for the mouse over the last 100 years enables researchers to manipulate and study gene function in vivo almost at will. Time and time again, scientific findings obtained through the use of mouse models have proven to be relevant to human health. The field of immunology in particular has profited tremendously from the powers of the mouse model and much of our knowledge of the workings of the immune system is derived from studies performed in mice.

Yet all that glisters is not gold. Researchers have used the mouse extensively as a model organism to study the pathogenesis of human infections and found that it imperfectly recapitulates many aspects of infectious disease as seen in patients. In fact, mice generally appear to be highly resistant to infections with human-specific pathogens like HIV, *Plasmodium falciparum*, and *Shigella flexneri*, especially if the pathogen is administered through the natural route of infection. Of course, the inherent resistance of mice to highly adapted human pathogens should not be a surprise: because pathogens co-evolve with and adapt to their preferred host, their successful specialization often renders them highly dependent on their host species [1]. Such host tropism or host restriction limits the usefulness of the mouse as a model for infectious disease research. One approach to overcome the limitations of the mouse model could be to genetically engineer mice that closely resemble humans in all those aspects relevant for host–pathogen interactions. To assess how realistic this ambitious goal may be, we must first understand the underlying molecular causes for host restriction.

## Species-Specific Immune Evasion by Pathogens Contributes to Host Tropism

For many infectious diseases, host restriction is at least in part based on the inability of a pathogen to colonize the non-typical host effectively. Colonization often relies on species-specific interactions of microbial ligands with host cell receptors. For example, the bacterial effector internalin A expressed by *Listeria monocytogenes* binds to the host E-cadherin receptor to mediate bacterial internalization, an essential step for the microbe to breach the intestinal epithelial barrier after oral ingestion [2]. A single amino acid change in the mouse ortholog of E-cadherin disrupts the interaction with internalin A and abrogates efficient bacterial invasion [2]. As a consequence, mice are relatively resistant to *L. monocytogenes* infections administered through the oral route. Transgenic mice expressing human E-caherin in the small intestine, on the other hand, are susceptible to oral infections with *L. monocytogenes* and develop enteropathogenicity and systemic infections, thus truthfully recapitulating some aspects of human disease [3].

Additionally, host restriction may be caused by the failure of pathogens to deter immune assaults in the non-typical host. For bacterial pathogens, this principle has been beautifully described for the human-restricted pathogen *Neisseria gonorrhoeae*, the causative agent of gonorrhea. Outer membrane porin molecules of *N. gonorrhoeae* can bind human but not rodent inhibitory molecules of the alternative and classical complement pathways, rendering gonococci resistant to complement-mediated killing by human serum but susceptible to rodent serum [4],[5]. In contrast, *Yersinia pestis*, a pathogen of both humans and rodents, binds complement inhibitory molecules of either host species and evades both human and rodent serum-mediated killing [4]. This example illustrates a general principle: a pathogen must be able to endure or overcome innate immune responses that drastically interfere with its survival and/or transmission to another suitable host. Pathogens must, therefore, have evolved subversion strategies for all the innate immune mechanisms that, if unrestricted, would result in their demise. A pathogen will, of course, acquire immune evasion strategies only against antimicrobial responses active in the host species with which it has co-evolved. Highly adapted human pathogens would therefore be vulnerable to an antimicrobial immune pathway present in mice but absent from humans. Recent work on IFNγ-activated host responses to intracellular pathogens has indentified a powerful cell-autonomous host defense system orchestrated by a family of GTPases named p47 Immunity Related GTPase (IRG proteins) that is present in mice but appears to be largely absent from humans and may exert limited antimicrobial functions in humans compared to mice [6],[7],[8],[9].

## In Mice IRG Proteins Are Essential for IFNγ-Induced Resistance towards Various Pathogens

Activation of the mammalian innate immune system by the cytokine IFNγ is essential for host resistance to many pathogenic organisms. Although IFNγ is produced by specialized immune cells, its receptors are found on nearly all cells, where it activates diverse responses that enable host cells to ward off intracellular infections by bacterial, viral, and protozoan pathogens [10],[11]. A few of the responses and their mediators are now well characterized, in particular the production of nitric oxide, the production of oxygen radicals, and the depletion of intracellular tryptophan stores by, respectively, inducible nitric oxide synthase, phagocyte oxidase, and indoleamine 2,3-dioxygenase (IDO) [10],[11]. However, it has been long known that these mechanisms cannot account for all the effects of the IFNγ response that result in cell-autonomous resistance to intracellular pathogens. Recently, this void in our knowledge has been partly filled with the discovery that several members of the IRG gene family exercise highly effective antimicrobial activities directed at a diverse set of bacterial and protozoan pathogens. Several research groups have used a gene knockout approach in mice to study the function of select representatives of the IRG family in host resistance. So far, mice lacking *Irgm1/Lrg-47*, *Irgm3/Igtp*, *Irgd*/*Irg-47*, and *Irga6/Iigp1* have been reported and shown to display some dramatic susceptibility phenotypes to several pathogens, highlighting the essential role of the murine IRG system in resistance to many infectious agents [12],[13],[14].

## Stark Differences Exist between the Murine and Human IRG Resistance Systems

**Figure 1:**
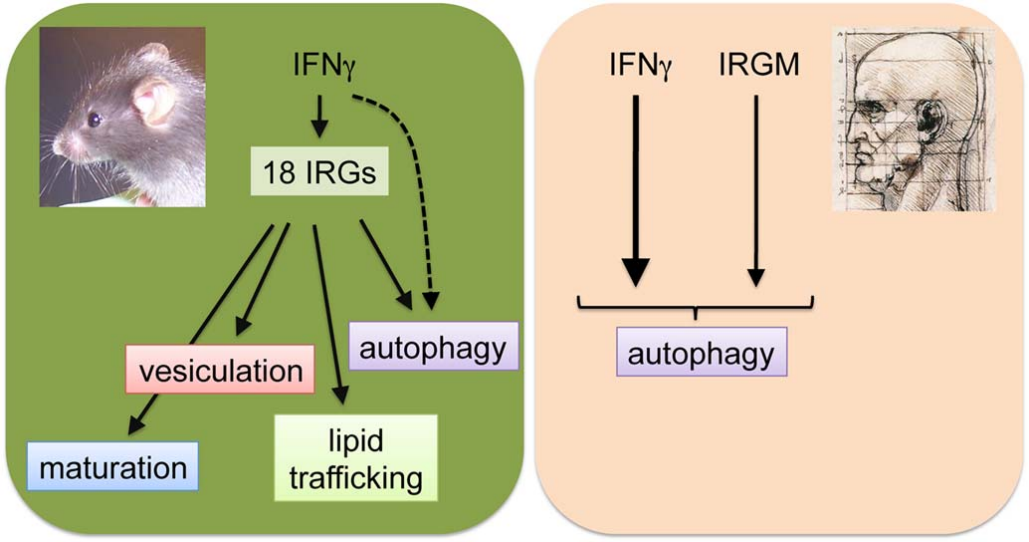
The Mouse and Human IRG Resistance Systems Are Distinct. In mouse cells as many as 18 IRGs are being induced upon IFNγ stimulation and mediate several antimicrobial activities, including the vesiculation of pathogen-containing vacuoles and accelerated maturation of phagosomes. Overexpression of mouse Irgm1 has also been shown to induce autophagy in macrophage-like RAW 264.7 cells [15], but so far no defect in autophagy in Irgm1 knockout macrophages has been reported. IFNγ is likely to induce autophagy also in an IRG-independent manner. In contrast to mouse IRGs, human IRGM is not IFNγ-inducible. IRGM has been shown to play a regulatory role in the execution of antimicrobial autophagy, but has not been associated with additional antimicrobial activities.

In light of the importance of IRG-mediated immunity in mice, it was surprising to find that an IFNγ-inducible IRG system appears to be lacking in humans. In contrast to mice, which express as many as 18 separate IRG genes upon IFNγ stimulation, the human genome possess only two IRG genes, *IRGC* and *IRGM*, neither of which is IFN-inducible [6]. The restricted expression pattern of IRGC, detected only in male gonads in both mice and humans, suggests that it does not play a universal role in the innate immune response [6]. IRGM, on the other hand, is constitutively expressed in a number of human cell lines. However, the severe truncations of IRGM protein compared to mouse IRG proteins suggests that the human IRGM must be functionally distinct from its mouse orthologs [6]. Therefore, it was surprising that both human IRGM and mouse Irgm1 were reported to play a role in the induction of autophagy, a multi-purpose cellular process with antimicrobial activity [15],[16],[17]. Notwithstanding some potential overlap in function, mouse IRGs but not human IRGM have been implicated in several additional antimicrobial activities other than autophagy, namely accelerated maturation of phagosomes, disintegration of pathogen-containing vacuoles through vesiculation of vacuolar membranes, and the modification of lipid trafficking (Figure 1) [13],[18],[19],[20]. Though a more careful analysis of the molecular activities of human IRGM will be required to draw any definitive conclusions, it seems as if the human IRG system has been stripped of most effector functions found in mice, with the exception of a regulatory role in autophagy. Whether any of these additional IRG-driven antimicrobial activities that exist in mice are lacking in human cells is currently not known. It may very well be that the immune functions embodied by IRGs in mice have been preserved in humans but are executed by a different set of molecules, a principle that is exemplified by the convergent evolution of NK inhibitory receptors in mice and humans [21]. In this context, it is worth mentioning that some of the most strongly induced proteins in IFNγ-activated cells belong to another GTPase family, the p65 guanylate binding proteins (GBPs), which are highly conserved between mice and humans [22]. Remarkably, it has recently been shown that GBPs like IRGs localize to the parasitophorous vacuolar membrane surrounding the protozoal pathogen *Toxoplasma gondii* [23]. Although the importance of this observation is presently unclear, it is suggestive of GBPs exerting immune effector functions that may be related to the activities that have been described for IRG proteins.

## IFNγ-Induced Resistance towards *Chlamydia trachomatis* Requires IDO-Dependent Tryptophan Depletion in Human Cells but IRG Responses in Mouse Cells

Strong support for the hypothesis that human IRGM exerts limited antimicrobial responses compared to the murine IRG system came from studies of the host response to the human intracellular bacterial pathogen *C. trachomatis*. In human cells, IFNγ-activated resistance to *C. trachomatis* depends predominantly on IDO-mediated tryptophan depletion [24],[25]. Supplementing growth media with tryptophan, thus specifically neutralizing the effect of IDO, completely reverses the growth inhibitory effect of IFNγ on *C. trachomatis* in most human cell lines in spite of detectable IRGM expression [6],[24]. Based on the assumption that IDO and IRGM act in distinct pathways, these results strongly suggest that IRGM does not exert IFNγ-dependent antimicrobial effects towards *C. trachomatis* in human cells. However, direct functional studies are required to decisively determine the role of IRGM in resistance to *C. trachomatis* infections in human cells. In contrast to human cells, most mouse cells do not induce IDO expression upon IFNγ stimulation [24],[26],[27], and IDO-deficient mice display wild-type resistance to *C. trachomatis* infections [20]. Instead, mice require at least three IRG genes, *Irgb10*, *Irgm1*, and *Irgm3*, for resistance to *C. trachomatis* infections both in vivo and in IFNγ-stimulated cells in vitro [28],[29]. Collectively, these studies show that the IRG system is essential for the innate immune response to *C. trachomatis* in mice, whereas IRGM appears to play no role in IFNγ-induced cell-autonomous resistance to *C. trachomatis* infections in many, if not all, human cells.

## Host-Adapted *Chlamydia* Strains Evade the IFNγ Response Specific to Their Respective Hosts

Because of the disparate effector functions of the human and mouse IFNγ response, it is expected that host-adapted pathogens for these two species should be equally divergent in their immune evasion strategies. In support of this hypothesis, we were recently able to show that the mouse-adapted strain *Chlamydia muridarum*, but not its close relative *C. trachomatis*, can specifically evade IRG-mediated host resistance by blocking access of IRG proteins to the *Chlamydia*-surrounding vacuolar membrane, the inclusion membrane [29]. On the other hand, and complementary to these results, *C. trachomatis* strains isolated from the human urogenital tract, but not *C. muridarum*, express tryptophan synthase, an enzyme capable of using exogenous indole for the synthesis of tryptophan [30],[31]. The ability of *C. trachomatis* to use indole (probably generated by the local microbial flora of the genital tract) provides *C. trachomatis* with a lifeline to endure IDO-mediated tryptophan starvation, and this is potentially a key element in the establishment of persistent infections in humans [30]. The divergent counterimmune mechanisms employed by the human pathogen *C. trachomatis* and the mouse-adapted pathogen *C. muridarum* clearly reflect the differences in the IFNγ responses of their respective hosts, a paradigm we expect to see recapitulated in other host–pathogen interactions.

## Do Other Mouse-Adapted Pathogens Counteract the IRG Response?

IRG genes are found throughout the vertebrate subphylum, but their representation is erratic: for instance, the genomes of zebrafish, rats, and dogs harbor IRGs, whereas the chicken genome is so far devoid of IRG genes [6]. We postulate here that pathogens adapted to IRG-deficient hosts (e.g., humans) are highly vulnerable to the antimicrobial effects of IRGs because these pathogens have not been under evolutionary pressure to acquire immune evasion strategies targeting IRG responses. Accordingly, the recent observation that the zoonotic pathogen *Chlamydia psittaci* is highly susceptible to IRG responses in mice [32] makes perfect sense given that birds, the natural host of *C. psittaci*, probably lack IRG genes [6]. Additionally, we postulate that intracellular vacuolar pathogens adapted to IRG-expressing hosts (e.g., mice) have evolved mechanisms to resist the IRG responses of their hosts, as demonstrated for *C. muridarum*. At a first glance this proposition seems to be in conflict with published work on *T. gondii*, a natural protozoal pathogen of mice, which has been shown to be susceptible to the IRG resistance system [12],[13],[14],[18]. However, in all of these studies a naturally occurring avirulent type II strain of *T. gondii* was used, while it has been recently shown that a virulent type I strain possesses an anti-IRG evasion strategy [33],[34]. Why does such an avirulent *T. gondii* strain exist? It seems likely on general grounds that IRG immune evasion by virulent *Toxoplasma* strains could decrease fitness of the pathogen because the IRG resistance system rescues the mouse from early mortality but does not prevent avirulent strains from establishing a chronic infection. In contrast, type I strains cause early death in mice in spite of a functional IRG system, thus reducing the likelihood of successful transmission of *Toxoplasma* to a new host. Since *Toxoplasma* is also a remarkably promiscuous pathogen, infecting many different intermediate hosts that are prey to cats, it should also be considered that not all its polymorphic variants are necessarily adaptations to successful colonization of mice. Nevertheless, it is clear that the IRG resistance mechanism is an important part of the co-adaptation between mice and *Toxoplasma*. In contrast, IRG-mediated effects play no part in human resistance to *Toxoplasma*, while resistance via tryptophan depletion caused by IFN-induced IDO has been well documented (reviewed in [35]). Indeed, the striking virulence difference between *T. gondii* type I strains on the one hand and types II and III strains on the other has been documented only in mice; in humans the clinical differences are subtle [36]. So far, *T. gondii* and *C. muridarum* are the only mouse-adapted pathogens that have been analyzed to some degree in relation to the IRG resistance mechanism. However, we expect that other rodent-adapted pathogens like *Y. pestis* or *Plasmodium berghei*, which have not been analyzed in this context, will also feature IRG evasion mechanisms. It would be of great interest to establish whether in general the presence of an efficient IRG resistance system in a species predicts a reduced IDO resistance system and vice versa. So far we have only humans and mice as the polar cases.

## Genetically Engineered Mice as Models to Study Human Infectious Disease

The host specificity of a pathogen arises from continued selection over thousands or millions of years for adaptation to the ecological niche provided by the host species. When a pathogen is introduced into a non-typical host (i.e., a host the pathogen has not co-evolved with), the acquired molecular fine-tuning is lacking. Consequently, the outcome of an encounter between a pathogen and a non-typical host differs in several aspects from the characteristics of an infection of a typical or natural host. Many combinations of host species with co-evolved pathogens lead to chronic infections with low pathogen load and inefficient establishment of immunity. In contrast, experimental infections of non-typical hosts often result in acute pathogenicity to the host, followed by complete clearance of the infection and rapid development of effective immunity after a single exposure [37]. Given the striking differences between human infections and the phenotypes displayed by mice exposed to the same human-adapted pathogens, it often becomes problematic to translate knowledge obtained in mouse studies to human diseases. Better animal models may provide a solution to this conundrum. Because chimpanzees are evolutionarily more closely related to humans, they are susceptible to many human pathogens and often display symptoms similar to those seen during infection of humans. Nonetheless, genetic differences still exist between humans and chimpanzees that affect host–pathogen interactions [4],[38],[39],[40],[41]. More importantly, there are ethical considerations as well as practical disadvantages inherent in primate models (e.g., high costs), which argue against the widespread use of these animal models in infectious disease research. Mouse models that more closely resemble human disease could serve as an alternative, though the establishment of such models will be difficult. Indeed, the construction of a *Mus homunculus* for research in immunobiology may appear to be a Sysiphean task: each barrier broken will reveal the next. Moreover, for every pathogen a different set of obstacles will have to be tackled. However, it may not be necessary to mimic faithfully every aspect of the human system in a mouse model as long as some features have been humanized that are relevant to the scientific question being asked. For instance, replacing the IRG-mediated resistance system with the IDO-mediated resistance system in urogenital epithelial cells using a gene-targeted mouse model should enable us to study the role of IFNγ-induced tryptophan depletion in the establishment of persistent *C. trachomatis* genital infections. Though the creation of humanized mouse models for infectious disease will require substantial effort and resources, the long-term benefits of these new models would undoubtedly be enormous.
